# Absolute configuration determination of pharmaceutical crystalline powders by MicroED *via* chiral salt formation^†^

**DOI:** 10.1039/d2cc00221c

**Published:** 2022-04-12

**Authors:** Bo Wang, Jessica F. Bruhn, Asmerom Weldeab, Timothy S. Wilson, Philip T. McGilvray, Michael Mashore, Qiong Song, Giovanna Scapin, Yiqing Lin

**Affiliations:** a Small Molecule Drug Product Development, Biogen, 115 Broadway, Cambridge, MA 02142, USA. bo.wang2@biogen.com; b NanoImaging Services, 4940 Carroll Canyon Road, San Diego, CA 92121, USA

## Abstract

Microcrystal electron diffraction (MicroED) has established its complementary role alongside X-ray diffraction in crystal structure elucidation. Unfortunately, kinematical refinement of MicroED data lacks the differentiation power to assign the absolute structure solely based on the measured intensities. Here we report a method for absolute configuration determination *via* MicroED by employing salt formation with chiral counterions.

Enantiomeric compounds have identical physicochemical properties in an achiral environment. However, they may trigger vastly different biochemical effects due to the stereo-selective nature of enzymes.^1^ Following the harsh lessons learned from thalidomide in the 1950s, the pharmaceutical industry has realized the essentiality of determining and controlling the absolute stereochemistry of any new chiral active pharmaceutical ingredient (API).^2^ While there are a number of experimental methods capable of determining the absolute configuration of chiral molecules, e.g. vibrational optical activity^3^ and NMR,^4^ single-crystal X-ray diffraction (scXRD) crystallography is the most widely used and trusted method due to its reliability of data interpretation.^5^

In X-ray diffraction, a resonance phenomenon known as anomalous dispersion causes peak intensities to differ between Bijvoet pairs.^6^ Such differences in Bijvoet pairs can be quantitatively reflected in the Flack parameter,^7–9^ which is widely used to identify the absolute structure of non-centrosymmetric crystals (hence the absolute configuration of chiral molecules). Although brighter X-ray sources and more sensitive detectors are now available, a crystalline sample still needs to be at least ~10 microns in the smallest dimension for scXRD. Thus, to obtain the absolute configuration of a new API early in drug development, it is necessary to grow a suitably large single crystal, a potentially daunting task.

As a testament to the challenges associated with growing large crystals, a technique involving soaking API molecules into a crystalline sponge has been developed^10^ and applied to the determination of the absolute configuration.^11^ Excitingly, the emergence of microcrystal electron diffraction (MicroED), a 3D electron diffraction (ED) technique, has allowed for the determination of structures of small molecules and proteins using sub-micrometre-sized crystalline samples.^12–16^ Pharmaceutical APIs may readily form crystalline powders suitable for MicroED, simplifying or even eliminating the need to perform crystallization screenings. The MicroED technique has the potential to unlock structural information for thousands of APIs for which growing large crystals is challenging.

Unfortunately, unlike X-ray diffraction, ED has not been demonstrated to produce significant anomalous signals, precluding the application of the Flack parameter in the determination of absolute structures from MicroED data.^16^ Two methods have emerged for the determination of absolute structures using ED data: using a combination of ED imaging coupled with atomic resolution crystal imaging for inorganic materials^17,18^ and applying dynamical refinement for small molecules.^19^ Unfortunately, collection of atomic resolution imaging mode images is not currently possible for radiation sensitive materials such as pharmaceutical APIs.^16^ Dynamical refinement of MicroED data is a promising approach, as dynamical scattering results from multiple scattering events, which are more prominent in ED compared to X-ray diffraction. As enantiomeric crystals produce different dynamical scattering profiles, these differences can be used to assign absolute structures.^19,20^ However, currently this approach has not been widely adopted due to the challenges of adaptation and implementing this new data processing workflow.

## Communication

Practically speaking, to obtain a structural model from MicroED data, it is highly desirable to process the data using the kinematical theory of diffraction, as opposed to the dynamical theory, which enables the convenience of using well-established data reduction software packages written for scXRD familiar to most crystallographers.^21^ In the absence of dynamical refinement, it is still possible to determine the absolute structure from MicroED data under certain conditions. For chiral crystals, two enantiomeric solutions are produced from diffraction data. Selection between these two potential solutions can be based on experimental measurements (anomalous dispersion for X-rays and atomic resolution images or dynamical effects for electrons) or prior knowledge of the sample. As crystallography produces only one pair of possible structures regardless of the number of chiral centers, knowledge of the configuration of one chiral center allows the assignment of the remaining chiral centers (relative configuration). It is therefore possible to assign the absolute configuration of an API molecule by introducing a chiral standard using salt and/or cocrystal formation, a well-known strategy used in scXRD^9,22,23^ and powder X-ray diffraction (PXRD).^24,25^ Adopting the same methodology, we expect that the absolute configuration of an API can be readily determined by MicroED using internally referenced chiral salt or cocrystal powders.

**Scheme 1:**
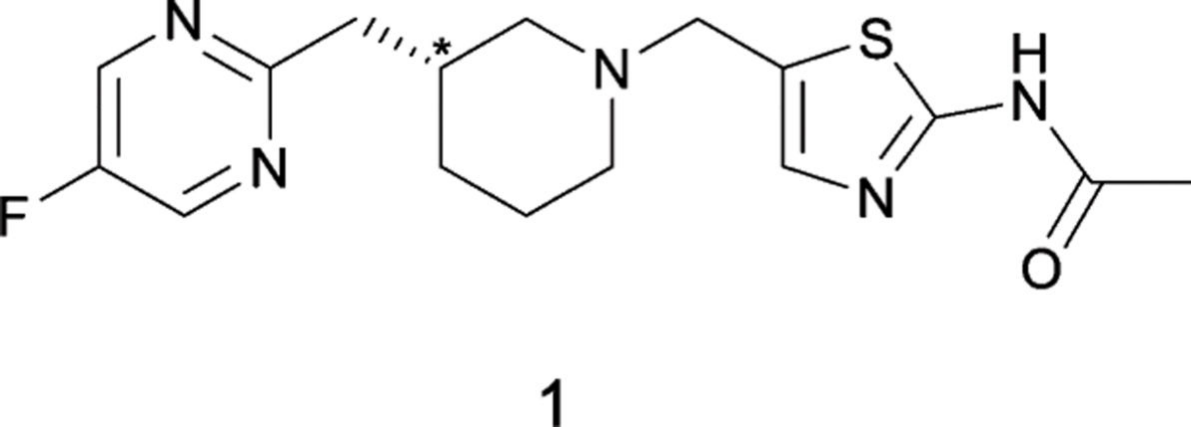
Chemical structures of API 1. The chiral center in question is indicated with a star.

In the early stages of pharmaceutical development, when the amount of material is limited and/or the API does not readily form large crystals suitable for scXRD, one may therefore consider MicroED to confirm both the molecular connectivity and the absolute configuration of an API. Any crystalline form of the API should be suitable for determining connectivity information and for chiral compounds, a minimal chiral salt/ cocrystal screening can produce crystalline powders suitable for determining the absolute configuration *via* MicroED. For the first time, we report the absolute configuration determination of micron-sized powders of a real-world pre-clinical drug candidate, API 1 (Scheme 1), by MicroED via chiral salt formation. Complementarily, the scXRD and MicroED structures of the free base form of API 1 were determined and compared.

**Fig. 1:**
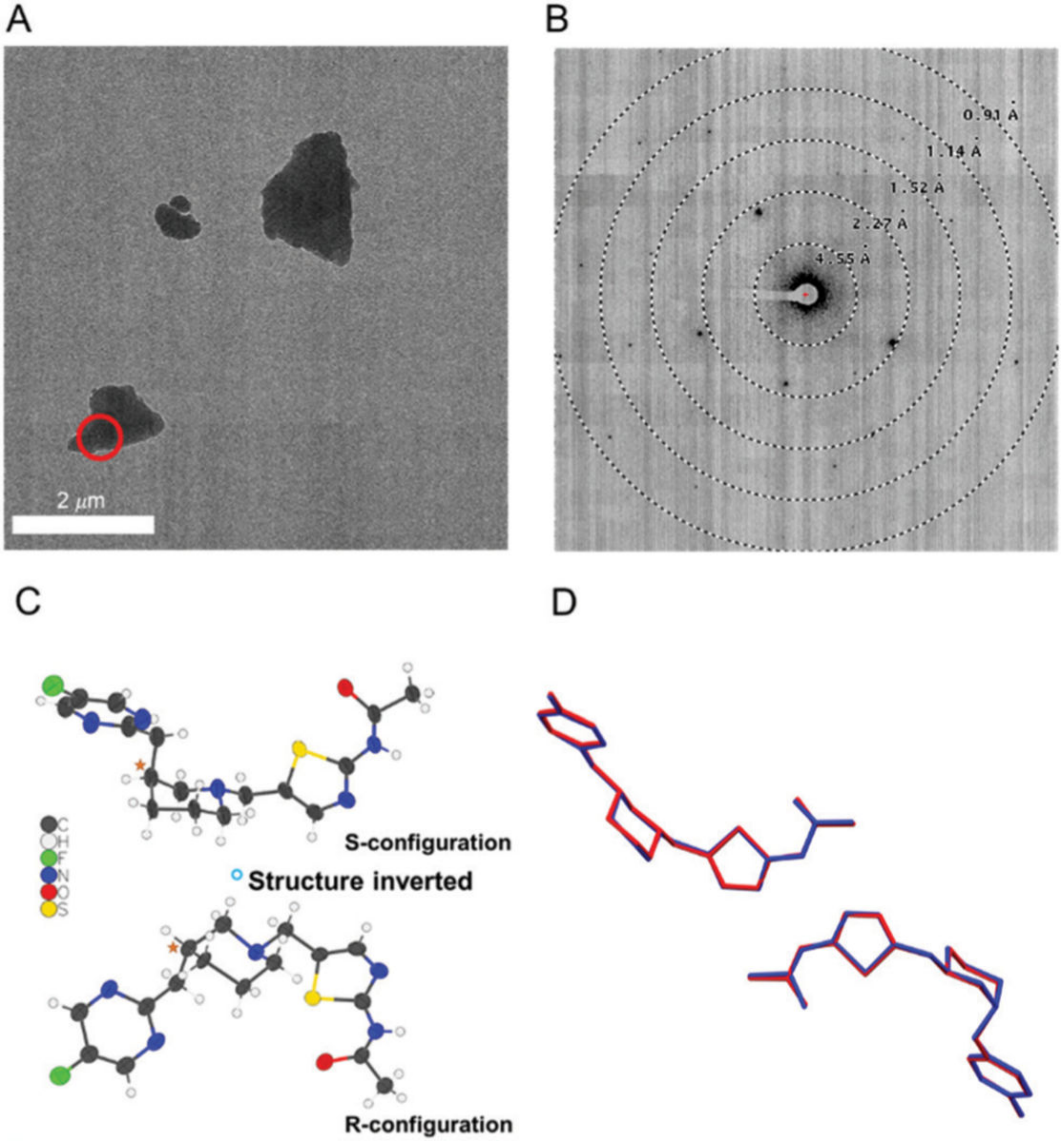
(A) TEM image of a microcrystal of API **1**. The target used for diffraction is shown in red. (B) The corresponding MicroED diffraction pattern. (C) One molecule of API **1** from the MicroED structure is shown in the two possible absolute configurations. Either configuration fits the experimental data equally well. (D) An overlay of the MicroED (blue) and scXRD (red) structures of API **1** in one asymmetric unit.

As rapid confirmation of molecular connectivity is critical for the drug development process, MicroED was explored for API 1. PXRD analysis of purified API 1 confirmed that this powdered sample was already highly crystalline without the need for further crystallization optimization. The crystal structure of the free base powder was then readily determined by MicroED using a pipeline method described previously (Fig. 1A and B).^21^ The free base form of API 1 crystallized in the chiral space group P1. The unit cell contains two enantiomerically pure molecules in different conformations. While the molecular connectivity was readily discerned from this structure, the absolute configuration could not be determined using kinematical refinement. Both enantiomeric structures fit the diffraction data equally well (Fig. 1C).

In parallel, a scXRD structure of the free base form of API **1** was determined for comparison purposes and to validate the MicroED-derived absolute configuration results. To obtain a suitably sized single crystal, a crystallization screening was required. Fortunately, a suitable single crystal sample (190 × 120 × 90 μm) was obtained by slow evaporation in tetrahydrofuran. The MicroED and scXRD structures represent the same free base form and are in excellent agreement. The root-mean-square deviation between these two structures for non-hydrogen atoms was 0.089 Å, highlighting the high quality of the MicroED structure (Fig. 1D).

API **1** contains a tertiary amine (pK_a_ ~ 7.6) that was predicted to form salts with acidic salt formers.^26^ To introduce an internal chiral standard into a crystalline form, a mini-salt screening was performed using four enantiomerically pure counterions: l/d-tartaric acids and l/d-malic acids. These salt forming counterions were chosen based on their pK_a_ values (a pK_a_ difference of greater than 3 between the API and the counterion is recommended^27^) and procurement considerations. Crystallization was carried out by precipitation of a stoichiometric mixture of API **1** and each counterion in organic solvents. Excitingly, this screening produced three microcrystalline hits. Among these hits, the D-malate salt of API 1 appeared to have the sharpest peaks in PXRD and was therefore chosen for MicroED structure determination.

**Fig. 2:**
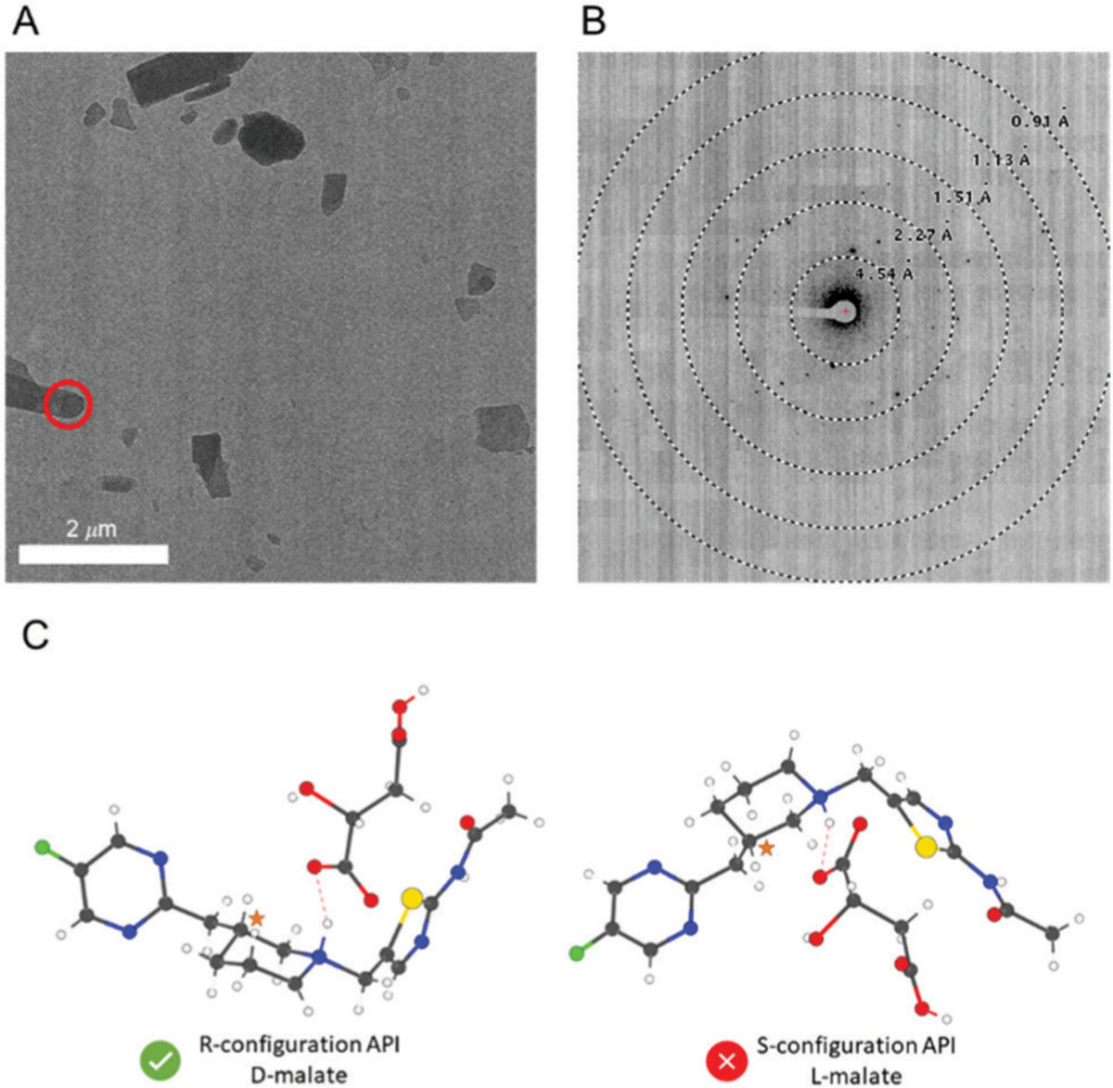
(A) TEM image of microcrystals of API **1**-D-malate. The target used for diffraction is shown in red. (B) The corresponding MicroED diffraction pattern. (C) The two potential configurations of the structure with the correct configuration indicated.

The D-malate salt form of API 1 diffracted to a lower resolution (1.02 Å) compared to the free base form (0.90 Å), but nevertheless, MicroED structure determination was still possible (Fig. 2). This salt crystalized in the chiral space group *P*2_1_ with two pairs of the API **1** cations and malate anions in the asymmetric unit. The molecular connectivity was as expected. As kinematical refinement was again performed, the inverted structural models (Fig. 2C) are equally correct based on the figure of merit from the MicroED data. However, because only the enantiomerically pure D-(+)-malic acid was used in producing this salt, the inverted crystal structure with the S-configuration API 1 and L-malate must be invalid. Therefore, the absolute configuration of the API 1 can be confidently concluded to be R. Supportively, this absolute configuration assignment was validated by the X-ray structure of the free base, with a Flack parameter of −0.008(5).^7^ We note that the D-malate salt crystals were too small for scXRD analysis and could only be determined by MicroED. As summarized in Table 1, our method provides an unambiguous assignment of the absolute configuration, with moderate sample preparation requirement and standardized data processing workflow.

In this work, we demonstrate the feasibility of absolute configuration determination of chiral APIs by MicroED *via* chiral salt formation. This approach can be instrumental for chiral compounds for which it is challenging to obtain large single crystals for scXRD experiments. This study relied on chiral salt formation, but we fully expect that this method could also be applied by employing chiral co-crystallization. With moderate sample preparation, rapid data processing, and most importantly, unambiguous result interpretation, this approach can serve as a powerful tool in a pharmaceutical scientist’s toolbox to determine the absolute configuration of a new **API** early in drug development, to ensure the quality of drug products, and ultimately, to improve the quality of life of patients.

BW conceptualized the project. BW, JB, and AW wrote the manuscript. AW performed the crystallization screening and characterized the samples prior to MicroED analysis. TW, PM, MM and QS prepared the grids and collected the MicroED data. JB processed the MicroED data and jointly refined the MicroED structures with BW. BW performed the scXRD work. YL and GS secured financial support and oversaw the project. All the authors contributed to the article and approved the submitted version.

NanoImaging Services is a commercial supplier of electron microscopy services to the biopharmaceutical and biotechnology industries.

## Supplementary Material

SI

## Figures and Tables

**Table 1:** Comparison between diffraction-based methods to assign the absolute configuration of chiral APIs

Technique	scXRD	PXRD Rietveld refinement	MicroED kinematical refinement	MicroED dynamical refinement
Sample requirement	– Large single crystal	– Crystalline powder with internal reference	– Crystalline powder with internal reference	– Crystalline powder
Advantage	– Reliability of results – Straightforward data processing		– Reliability of results	– Minimal sample preparation
Challenges	– Crystal growth	– Difficulty in solving complexstructures – Introduction of chiral reference	– Introduction of chiral reference	– Requires ED-specific data processing
