# Supplementary material for: Absolute configuration determination of pharmaceutical crystalline powders by MicroED via chiral salt formation
Source: Chem Commun (Camb). Author manuscript; Available in PMC 2022 Apr 14. (PMC9004345; doi:10.1039/d2cc00221c)
Supplement: SI [file NIHMS1792239-supplement-SI.pdf]

Electronic Supplementary Material (ESI) for ChemComm.
This journal is © The Royal Society of Chemistry 2022

SUPPORTING INFORMATION

Absolute Configuration Determination of Pharmaceutical Crystalline Powders by MicroED via Chiral Salt Formation

Bo Wang[1*†], Jessica F. Bruhn[2†], Asmerom Weldeab[1], Timothy S. Wilson[2], Philip T. McGilvray[2], Michael Mashore[2], Qiong Song[2], Giovanna Scapin[2], and Yiqing Lin[1]

Abstract: Microcrystal electron diffraction (MicroED) has established its complementary role alongside X-ray diffraction in crystal structure elucidation. Unfortunately, kinematical refinement of MicroED data lacks the differentiation power to assign the absolute structure solely based on the measured intensities. Here we report a method for absolute configuration determination via MicroED by employing salt formation with chiral counterions.

DOI: 10.1039/x0xx00000x

Table of Content

1. Materials 3

2. Crystalline Sample Preparation 3

2.1 Mini Salt Screening 3

2.2 Synthesis of API 1-D-malate 3

2.3 Single Crystal Growth of API 1 Free Base 3

2.4 Characterization 4

2.4.1 Powder X-ray Diffraction (PXRD) 4

2.4.2 Polarized Light Microscopy (PLM) 4

2.4.3 Thermogravimetric Analysis (TGA) and Differential Scanning Calorimetry (DSC) 5

2.4.4 Proton Nuclear Magnetic Resonance (^{1}H-NMR) 6

3. MicroED Structure Determination 7

4. scXRD Structure Determination 8

5. Structure Detail 9

5.1 API 1 Free Base Form Structure 9

5.2 API 1-D-malate Structure 10

6. References 11

1. Materials

API **1** (>98.5% purity), (R)-N-(5-((3-((5-fluoropyrimidin-2-yl)methyl)piperidin-1-yl)methyl)thiazol-2-yl)acetamide, was internally developed. D-(+)-Malic acid (>98.0% purity) was purchased from TCI. L-(-)-malic acid (≥95% purity), L-(+)-tartaric acid (>99% purity), D-(-)-tartaric acid (>99% purity), isopropyl acetate (>98% purity), acetone (>99.5% purity), and tetrahydrofuran (>99.9% purity) were purchased from Sigma-Aldrich. All materials were used without further purification.

2. Crystalline Sample Preparation

2.1 Mini Salt Screening

Salt formers (D-malic acid, L-malic acid, D-tartaric acid and L-tartaric acid) were selected based on pKa and procurement considerations. Screening solvents (isopropyl acetate and acetone) were chosen based on solubility considerations. The mini salt screen was composed of eight experiments in which API **1** was mixed with each of the four acids in either isopropyl acetate or acetone. Screening was carried out using ~10 mg API **1** for each test condition. Solutions of the salt formers were heated and then added to solutions of API 1 at 1.1 to 1 molar equivalents and stirred at room temperature. White precipitate formed immediately for all conditions tested. The solids were then filtered and characterized by powder X-ray diffraction (PXRD), see **Figure S1**. As sufficient material was desired for analysis by techniques other than MicroED, the crystallization reaction for the best hit as identified by PXRD (D-malate salt obtained from isopropyl acetate) was scaled up as described below.

2.2 Synthesis of API 1-D-malate

50 mg of API **1** was dissolved in 3.0 ml isopropyl acetate in an 8 mL vial at room temperature. In a separate vial, 21.1 mg (1.1 molar equivalents) of D-malic acid was dissolved in 1.0 ml of isopropyl acetate at 45 °C. The heated D-malic acid solution was added dropwise to the API **1** solution while stirring at room temperature. A white precipitate appeared immediately, and the mixture continued to be stirred for 2 hours at room temperature. The precipitate was then filtered using Büchner funnel, producing a white cake-like solid. The cake was ground using a mortar and pestle, and the resulting powder was placed in a 40 °C oven overnight to remove any residual solvent. Subsequently, full characterization of the D-malate salt (**Scheme S1**) was performed by PXRD, PLM, DSC, TGA, ^{1}H-NMR as shown below.

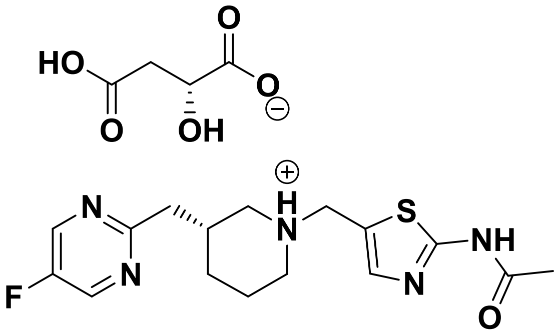

API 1-D-malate

Scheme S1. Chemical structure of API **1**-D-malate.

2.3 Single Crystal Growth of API 1 Free Base

4.5 mg of API **1** was dissolved in 200 μL tetrahydrofuran in a the 9-well glass plate. The clear solution was covered with Parafilm. Several holes were punctured in the film via a syringe needle to allow the solution to evaporate slowly. Single crystals were obtained and immersed into Paratone® N oil for surface cleaning and mounting.

2.4 Characterization

2.4.1 Powder X-ray Powder Diffraction (PXRD)

Crystallinity of the API **1** salts were studied using an XRD-D8 X-ray powder diffractometer using Cu Kα radiation (Bruker, Madison, WI). The instrument was equipped with a long, fine focus X-ray tube. The tube voltage and amperage were set to 40 kV and 40 mA, respectively. The divergence slit was set at V10.0 mm mode, with K_b filter and 2.5° AxialSoller inserted. Diffracted radiation was detected by a LYNXEYE (1D mode) detector. A 2θ continuous scan from 3.00 to 39.99° at 0.016° increment was taken (2287 steps with 0.5 sec exposure time). The sample was measured using a zero-background plate. The sample pan rotation speed was set to 15 rotations/min.

From the PXRD traces (**Figure S1**), three of the four conditions appeared to have formed crystals while API **1**-tartrate appears to be amorphous. Furthermore, API **1**-D-malate appeared to have the sharpest peaks, indicating a higher degree of crystallinity. It was for this reason that API **1**-D-malate was selected for MicroED data collection.

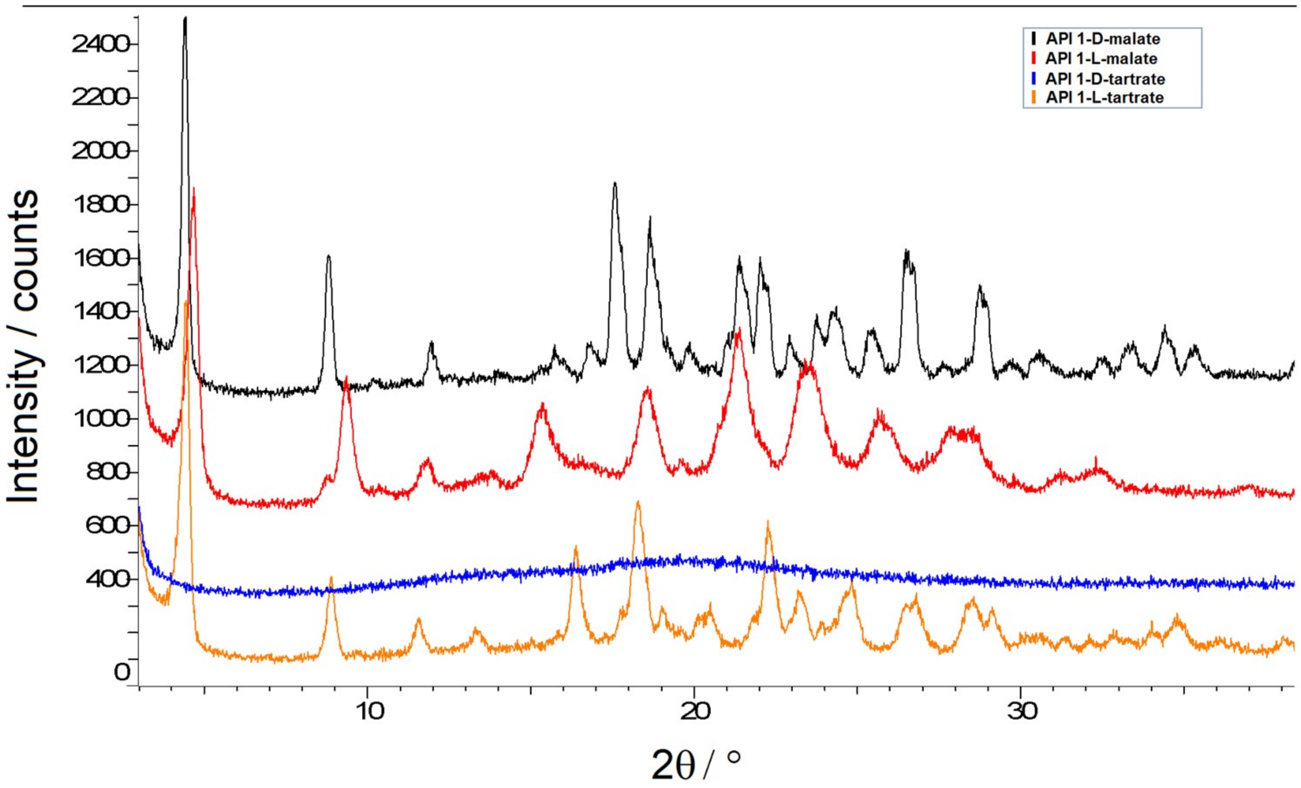

Figure S1. PXRD of the API **1** salts formed in isopropyl acetate. The diffraction patterns indicate that all formulations were crystalline except for API **1**-D-tartrate, which appears to be amorphous. Among the three crystalline hits, API **1**-D-malate appears to be the most crystalline as indicated by the sharper peaks. Note that the API **1** salts formed in acetone shows the same result, with wider diffraction peaks.

2.4.2 Polarized Light Microscopy (PLM)

Optical microscopic image analysis was performed using an Olympus BX51 microscope (Olympus Corp, Japan) equipped with a QImaging Micropublusher 5.2 RTV camera (QImaging, Inc., Canada), and imaging capturing and processing software QCapture Pro, version 6.0.0.412 by Media Cybernetics, Inc. and QImaging, Inc.
*API **1**-D-malate:* As shown in Figure **S2a**, the D-malate salt is a fine powder with birefringence. The crystal dimensions are on the order of 1-10 microns.

*API **1** free base:* As shown in Figure **S2b**, the free base material obtained directly from the vendor is a fine powder with birefringence. The crystal dimensions are below 50 microns.
*API **1** free base single crystal:* As shown in Figure **S2c**, the single crystal of the free base has birefringence. The parallelepiped-shaped crystal dimensions are on the order of 50 microns.

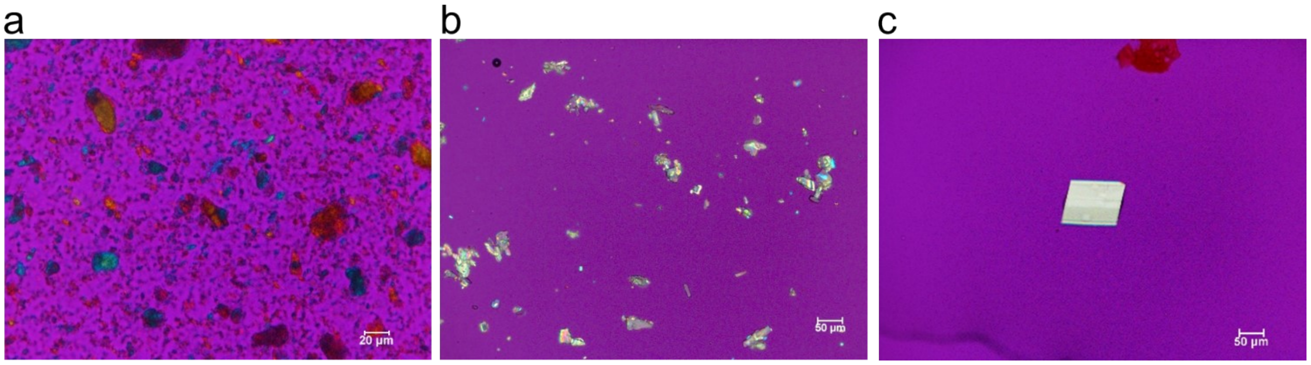

Figure S2. PLM images of API **1** crystalline samples: (a) API **1**-D-malate crystalline powder used for MicroED data collection; (b) API **1** free base crystalline powder, obtained internally without recrystallization, and used for MicroED data collection; (c) The API **1** free base single crystal sample used for scXRD data collection.

2.4.3 Thermogravimetric Analysis (TGA) and Differential Scanning Calorimetry (DSC)

Thermal properties of the putative D-malate salt were examined using a Discovery Thermogravimetric Analyzer (TGA, TA Instruments) and a Discovery Differential Scanning Calorimeter (DSC, TA Instruments). Approximately ~2mg of API **1**-D-malate was placed in a closed aluminum pan for DSC analysis and in an open aluminum pan for TGA analysis. The thermal analysis was performed with a linear gradient from 25°C to 300°C at 10°C/min for both the TGA and DSC studies. As shown in **Figure S3**, no weight loss was observed by TGA until at least 160°C, which indicates the anhydrous/unsolvated nature of the crystal form. DSC indicated that the D-malate salt form of API **1** has a melting onset at 164°C.

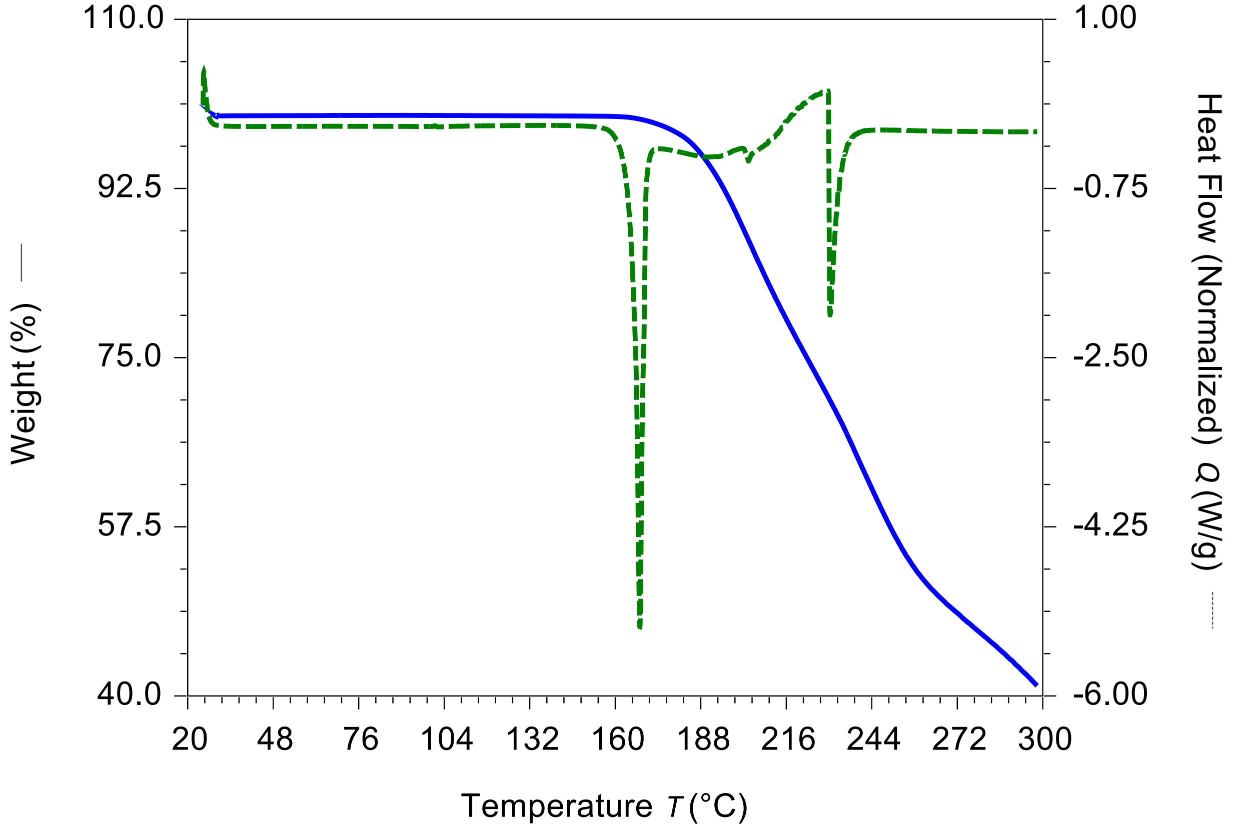

Figure S3. Overlayed TGA and DSC traces of API **1**-D-malate.

2.4.4 Proton Nuclear Magnetic Resonance (^{1}H-NMR)

Formation of the D-malate salt was confirmed by proton nuclear magnetic resonance (^{1}H-NMR), which indicated a 1:1 stoichiometry between API **1** and D-malic acid (**Figure S4**). The ^{1}H-NMR spectra of API **1**-D-malate was acquired on a Bruker-Biospin AVANCE NMR spectrometer operating at 500 MHz. Chemical shifts (δ) were based in ppm and multiplicities were reported as follows: s = singlet, bs = broad singlet, d = doublet, dd = doublet of doublet, t = triplet, q = quartet, m = multiplet. Sample was collected in DMSO-*d6* and the spectrum is consistent with structure based upon chemical shift. δ: **11.98 (bs, 1H), 8.78 (s, 2H), 7.26 (s, 1H), 4.18 (t, 1H), 3.74 (q, 2H), 2.85-2.74 (m, 4H), 2.59 (dd, 1H), 2.41 (dd, 1H), 2.21-2.05 (m, 5H), 2.02-1.91 (m, 1H), 1.70-1.54 (m, 2H), 1.50-1.38 (m, 1H), 1.06-0.95 (m, 1H).**

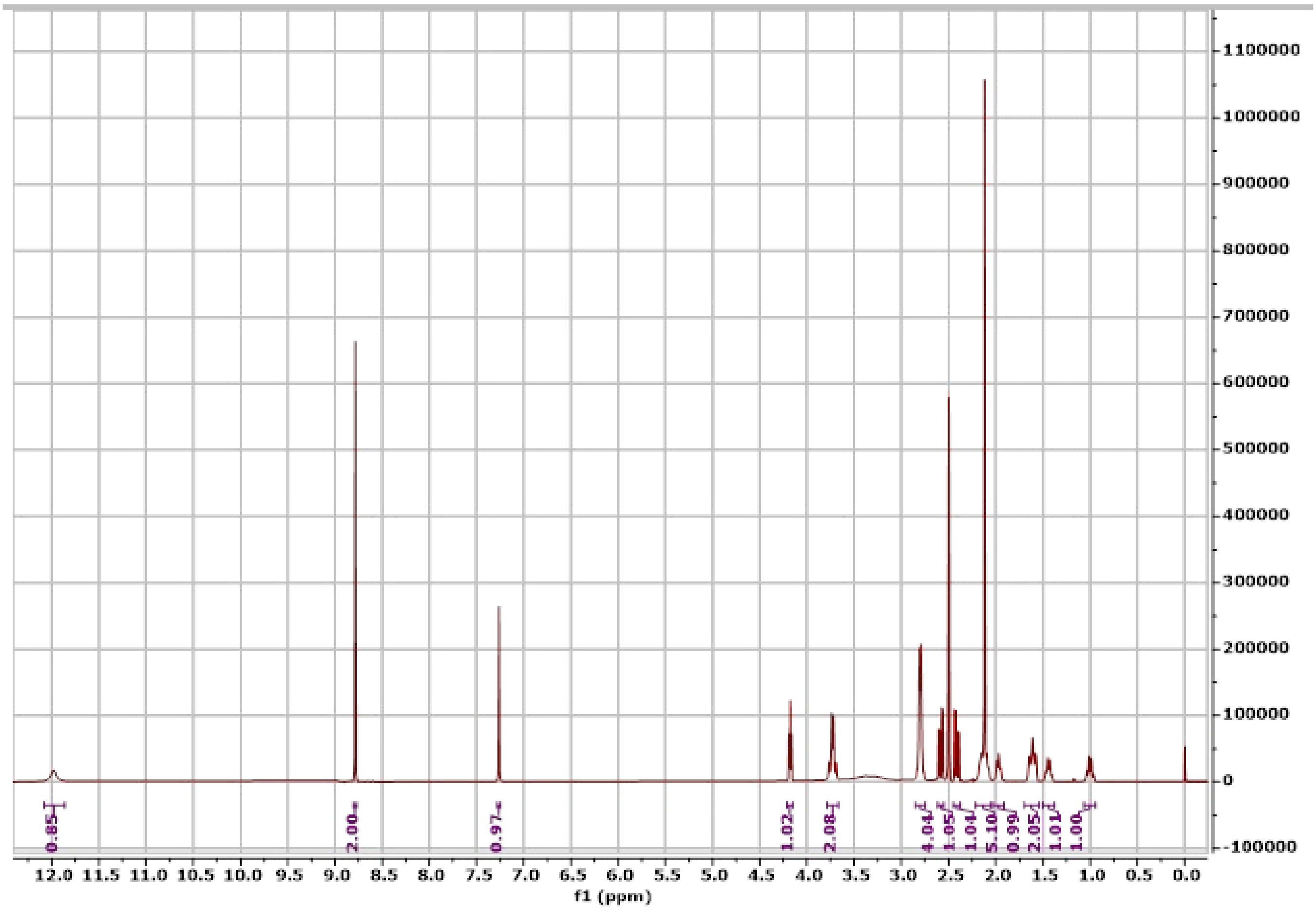

Figure S4. ^{1}H-NMR spectrum of API **1**-D-malate.

3. MicroED Structure Determination

*API **1** free base:* A small amount of powdered material was lightly grounded between two glass slides and applied to a pure carbon grid (Ted Pella 01840). MicroED data was collected as previously described using a Thermo Fisher Scientific (Hillsboro, OR, United States) Glacios Cryo Transmission Electron Microscope equipped with a CETA-D detector[1,2]. Datasets were indexed, refined, integrated and scaled using the program DIALS (Diffraction Integration for Advanced Light Sources, Copyright © 2015 Diamond Light Source, Lawrence Berkeley National Laboratory and the Science and Technology Facilities Council)[3]. Eleven datasets were collected, and the best nine datasets were combined to increase completeness and redundancy. Xia2 was used to convert file types[4] and XPREP[5] was used to analyze possible space groups. The data were phased by dual space methods with SHELXD[6]. Kinematical refinement was carried out using SHELXL[7] as implemented in Olex2[8] and validation was carried out in PLATON[9]. All hydrogen atoms were refined as riding models with fixed U_{iso} 1.2 or 1.5 (terminal methyl) times of the heavy atom attached. We note that the EXTI parameter was not used during refinement. Raw data is available on Zenodo (10.5281/zenodo.5799657). The final structure and reduced reflections can be accessed from the CCDC (2130868). Selected crystallographic data is summarized in **Table S1**.

*API **1**-D-malate:* A small amount of powdered material was applied directly to a pure carbon grid (Ted Pella 01840) and the grid was heated in a vacuum oven at 40°C for 15 minutes to remove volatile contaminants. MicroED data was collected as previously described[1,2]. Three hundred and twenty-one datasets were collected using automated methods and the best ten datasets were combined to increase completeness and redundancy. Data were processed as described above. The asymmetric unit appears to contain two copies of API **1** and two D-malates. One of the two D-malate counterions in the asymmetric unit was too disordered to model confidently. Instead, a solvent mask was employed using Olex2 to account for the disordered D-malate. As one D-malate was still clearly resolved, relative configuration could still be established. Due to the low resolution of the data, various constraints and restraints were employed to idealize the structure. All hydrogen atoms were refined as riding models with fixed U_{iso} 1.2 or 1.5 (terminal methyl) times of the heavy atom attached. We note that the EXTI parameter was not used

during refinement. Raw data is available on Zenodo (10.5281/zenodo.5803545). The final structure and reduced reflections can be accessed from the CCDC (2132512). Selected crystallographic data is summarized in **Table S1**.

4. scXRD Structure Determination

Single crystal X-ray diffraction data from API **1** free base was collected at 100 K using a Rigaku Synergy-S single crystal diffractometer (Cu*K*a radiation, λ = 1.54184 Å). The instrument was equipped with the HyPix-6000HE hybrid photon counting detector and the Oxford Cryostream 800 cryogenic system. The X-ray tube was operated at 50 kV and 1 mA. Cell parameters and an orientation matrix for data collection were retrieved and refined (least-squares refinement) by *CrysAlisPro* (*Rigaku*, *V171.41.98a*) software package[10]. The dataset was collected to a minimum 2θ of 6.73° and a maximum 2θ of 155.1°.

The x-ray diffraction frames were integrated with the CrysAlisPro (Rigaku, V171.41.98a) software package[10]. A total of 66717 reflections were collected, of which 6654 reflections were unique. The final cell parameters at 100.00(10) K were refined with 49730 strong reflections. Lorentz correction, polarization correction, and empirical absorption corrections (spherical harmonics) were performed using CrysAlisPro (Rigaku, V171.41.98a)[10]. The initial structure was solved in Olex2[8] using ShelXT[11] using Intrinsic Phasing and refined with ShelXL[7] using full-matrix least-squares on F^2. In the final model, no constraints and restraints were applied, except that all hydrogen atoms were refined as riding models with fixed U_{iso} 1.2 or 1.5 (terminal methyl) times of the heavy atom attached. Selected crystallographic data is summarized in **Table S1**. Note that the Flack parameter was found to be -0.008(5), showing that the absolute structure was correctly assigned.

Table S1. Crystallographic data summary.

Data Collection, Reduction and Refinement Information			
	**API 1 Free Base**	**API 1 Free Base**	**API 1 - D-Malate**
CCDC	2130868	2130542	2132512
Data Collection			
Wavelength (Å)	0.025 (electrons, FEG)	1.54 (X-rays, Cu Kα)	0.025 (electrons FEG)
Crystal Size (μm)	~ 1.5 x 0.5 x 0.2	190 × 120 × 90	~ 2 x 1 x 0.2
Temperature (°C)	-193	-173	-193
Camera length/Detector Distance (mm)	1100	34	1100
Oscillation per frame (°)	0.89	0.5	0.89
Dose per degree (e^-/Å^2)	~0.028	-	~0.028
Data Reduction			
Space group	*P*1	*P*1	$P2_1$
Number of datasets comb.	9	1	10
Unit cell lengths a, b, c (Å)	6.03, 10.90, 13.52	6.02, 10.99, 13.55	9.24, 6.01, 40.58
angles α, β, γ (°)	75.67, 84.27, 78.20	76.04, 84.61, 78.55	90, 94.35, 90
Resolution (Å)[a]	13.08-0.90 | 13.08-2.44 | 0.92-0.90	13.12-0.79 | 13.12-1.72 | 0.82-0.79	40.47-1.02 | 40.50-2.77 | 1.04-1.02
Total Reflections[a]	21417 | 1180 | 657	66717 | 8348 | 1422	22379 | 1254 | 790
Unique Reflections[a],[b]	2298 | 119 | 98	3569 | 356 | 365	2334 | 147 | 91
Multiplicity[a],[b]	9.3 | 9.9 | 6.7	18.7 | 23.4 | 3.9	9.6 | 8.5 | 8.7
Completeness (%)[a],[b]	95.0 | 96.0 | 92.5	98.4 | 100 | 86.5	94.1 | 99.3 | 89.2
I/σ(I) [a],[b]	5.1 | 21.0 | 0.3	64.0 | 92.0 | 32.0	4.8 | 33.8 | 0.2
R_{meas}[a],[c]	0.30 | 0.14 | 4.70	-	0.32 | 0.12 | 2.06
R_{pim}[a],[c]	0.09 | 0.05 | 1.71	-	0.13 | 0.05 | 0.85
$CC_{1/2}$[a]	0.98 | 0.98 | 0.19	-	0.99 | 1.00| 0.15
R_{int}	0.256	0.038	0.281
R_{sigma}	0.317	0.019	0.495
Refinement			
Chemical Formula	$C_{16}H_{20}FN_5OS$	$C_{16}H_{20}FN_5OS$	$C_{16}H_{21}FN_5OS$ • $C_4H_5O_5$[d]

Z	2	2	4
Z'	2	2	2
Data | Restraints | Param.	4510 | 315 | 435	6654 | 3 | 436	4190 | 375 | 494
R_1 | wR_2 for I≥2σ(I)	14.8% | 32.4%	2.6% | 6.8%	12.2% | 28.1%
R_1 | wR_2 for all data	22.9% | 35.1%	2.6% | 6.8%	22.9% | 30.6%
GooF (Goodness of Fit)	0.94	1.09	0.81
Flack Parameter	-	-0.008(5)	-

[a]Data reduction statistics are broken up into three resolution bins: Overall, Low and High.

[b]Unique reflections, multiplicity, completeness and I/σ(I) were calculated by combining Friedel pairs (I).

[c]R_{meas} and R_{pim} were calculated without combining Friedel pairs (I+/-).

[d]Half of the D-malate ($C_4H_5O_5$) contents of this crystal is ordered and modeled, while the other half was accounted for using solvent masking in Olex2.

5. Structure Details

5.1 API 1 Free Base Form Structure

The crystal structures of the free base form of API **1,** determined from the MicroED experiment (80.15 K) and scXRD experiment (100.0 K), are nearly identical. A structure similarity calculation of these two structures was performed using "Crystal Packing Similarity" function in the CSD-Materials module in Mercury 4.3.0.[12] The high similarity between these two crystal structures from these two diffraction techniques is reflected in the low root mean square deviation (RMSD) of the two structures (**Figure 1D**). Overlaying and comparing the two unique API **1** molecules in one unit cell from both techniques yielded a RMSD of 0.089 Å without hydrogens. Furthermore, the calculated PXRD patterns derived from the two structures have a high similarity score of 0.99794. Notably, the thermal ellipsoids of the atoms obtained from the MicroED structure are slightly larger, compared with the scXRD structure, possibly due to dynamical effects which were not accounted for during refinement. The inverted free base MicroED structure shown in **Figure 1C** was generated using "MOVE 1 1 1 -1" command implemented in SHELXL.[7]

It worth noting that within the unit cell of the free base from of API **1**, two unique API **1** molecules are tethered by two ideal hydrogen bonds between pairs of amines (N5A-H5A…N4B and N5B-H5B…N4A, **Figure S5**). In the scXRD structure, the donor/acceptor distances, and the angles of the two hydrogen bonds are 2.918 Å (176.78°) and 2.903 Å (176.34°), respectively. Also, the two unique API **1** molecules adopt different conformations, partially due to the geometric flexibility of the saturated six membered ring bearing the chiral amine. This can be easily seen in the overlay of the two molecules, as shown in **Figure S6**.

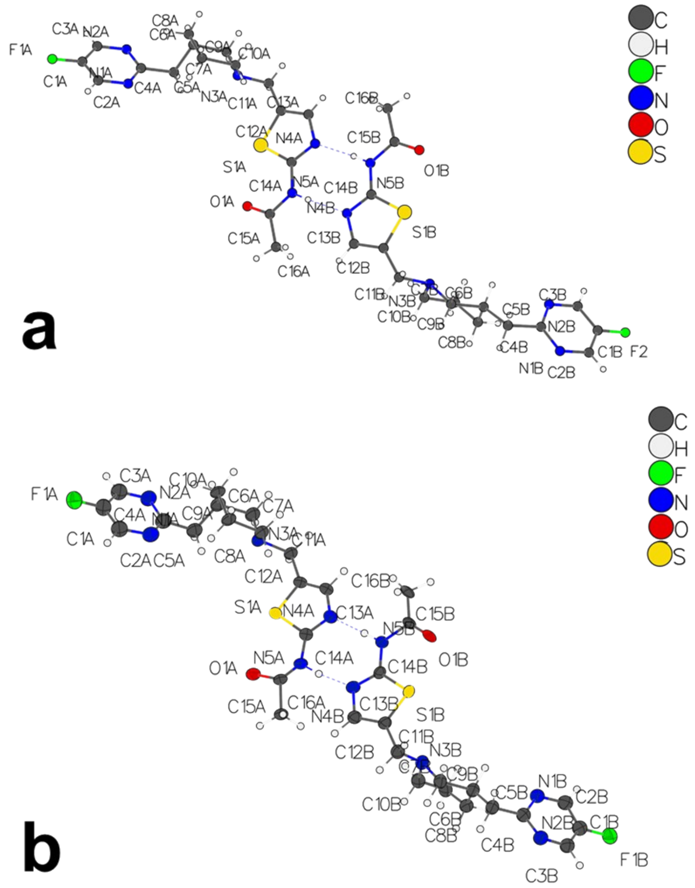

Figure S5. Crystal structure of free base form of API **1**. One asymmetric unit (unit cell) contains two API **1** molecules tethered by two pairs of hydrogen bonds: (a) scXRD structure; (b) MicroED structure; The ORTEP diagram (50% probability level) is drawn with Olex2[8]

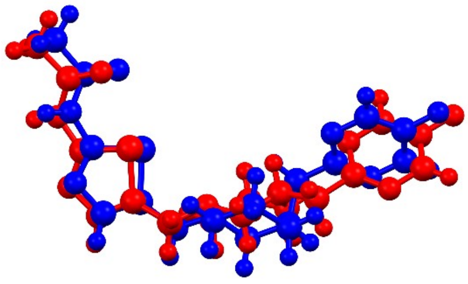

Figure S6. Overlay of the two API **1** molecules within the unit cell (using the scXRD structure), adopting different conformations. The ball-and-stick model is drawn with Mercury.[12]

5.2 API 1-D-malate Structure

The API **1**-D-malate salt structure determined by MicroED is depicted in **Figure S7**. One asymmetric unit of API **1**-D-malate contains two pairs of API **1** cations and D-malate anions, though only one D-malate could be confidently modeled. The remaining D-malate was too disordered to model and was therefore accounted for using solvent masking as implemented in Olex2. As one copy of D-malate could still be clearly modeled, the absolute configuration of API **1** could be inferred relative to the single D-malate. The acidic hydrogens of the D-malic acids

are modelled as deprotonated while the tertiary amine groups of API **1** are modeled as protonated. The distance of the salt bridge between N3A and O1C is 2.75 Å and 2.77 Å. The inverted structure shown in **Figure 2C** was made using "MOVE 1 1 1 -1" command implemented in SHELXL.[7]

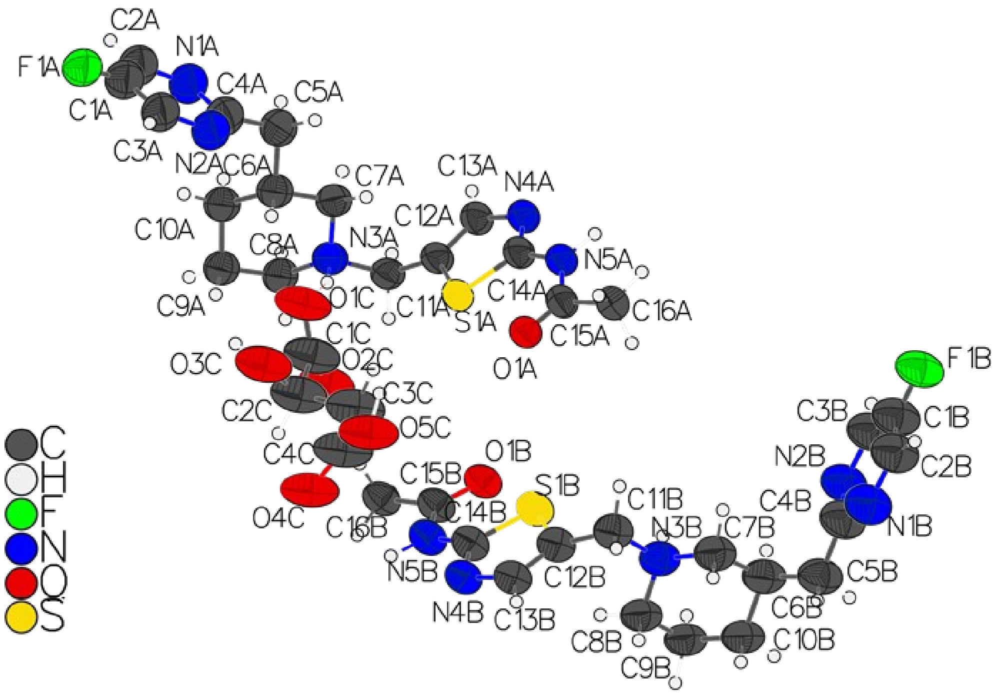

Figure S7. One asymmetric unit of API **1**-D-malate contains two pairs of API **1** cations and D-malate anions. One of the D-malates was too disordered to accurately model and was instead modeling using solvent masking, which is why it is not pictured above. The acidic hydrogens of the D-malic acids are modelled to protonate the tertiary amine groups of the API **1** molecules. The ORTEP diagram (50% probability level) is drawn with Olex2.[8]

6. References

1 J. F. Bruhn, G. Scapin, A. Cheng, B. Q. Mercado, D. G. Waterman, T. Ganesh, S. Dallakyan, B. N. Read, T. Nieusma, K. W. Lucier, M. L. Mayer, N. J. Chiang, N. Poweleit, P. T. McGilvray, T. S. Wilson, M. Mashore, C. Hennessy, S. Thomson, B. Wang, C. S. Potter and B. Carragher, *Front. Mol. Biosci.*, 2021, **8**, 354.
2 A. Cheng, C. Negro, J. F. Bruhn, W. J. Rice, S. Dallakyan, E. T. Eng, D. G. Waterman, C. S. Potter and B. Carragher, *Protein Sci. Publ. Protein Soc.*, 2021, **30**, 136–150.
3 M. T. B. Clabbers, T. Gruene, J. M. Parkhurst, J. P. Abrahams and D. G. Waterman, *Acta Crystallogr. Sect. Struct. Biol.*, 2018, **74**, 506–518.
4 G. Winter, *J. Appl. Crystallogr.*, 2010, **43**, 186–190.
5 Sheldrick, G.M. (2008) XPREP Version 2008/2. Bruker AXS Inc., Madison.
6 T. R. Schneider and G. M. Sheldrick, *Acta Crystallogr. D Biol. Crystallogr.*, 2002, **58**, 1772–1779.
7 G. M. Sheldrick, *Acta Crystallogr. Sect. C Struct. Chem.*, 2015, **71**, 3–8.
8 O. V. Dolomanov, L. J. Bourhis, R. J. Gildea, J. A. K. Howard and H. Puschmann, *J. Appl. Crystallogr.*, 2009, **42**, 339–341.
9 A. L. Spek, *J. Appl. Crystallogr.*, 2003, **36**, 7–13.
10 CrysAlisPRO, Oxford Diffraction /Agilent Technologies UK Ltd, Yarnton, England.
11 G. M. Sheldrick, *Acta Crystallogr. Sect. Found. Adv.*, 2015, **71**, 3–8.
12 C. F. Macrae, P. R. Edgington, P. McCabe, E. Pidcock, G. P. Shields, R. Taylor, M. Towler and J. van de Streek, *J. Appl. Crystallogr.*, 2006, **39**, 453–457.
